# Prevalence of CCR5 Delta 32 Genetic Variant in the Turkmen Population of Golestan Province, Northeast of Iran

**DOI:** 10.1155/2023/8823863

**Published:** 2023-06-21

**Authors:** Elmira Norasi, Mostafa Rastegar, Seyedeh Delafruz Hosseini, Bahman Aghcheli, Alireza Tahamtan

**Affiliations:** ^1^School of International, Golestan University of Medical Sciences, Gorgan, Iran; ^2^Department of Microbiology, Faculty of Medicine, Golestan University of Medical Sciences, Gorgan, Iran; ^3^Infectious Diseases Research Center, Golestan University of Medical Sciences, Gorgan, Iran

## Abstract

The 32 bp deletion in the chemokine receptor (C-C motif) 5 gene (CCR5*Δ*32) is a natural loss of function polymorphism that prevents the protein from locating on the cell surface. This genetic variation acts as a double-edge sword in the pathogenesis/defense mechanism of different health conditions, such as viral infections, autoimmune diseases, and cancers. Here, we evaluated the prevalence of the CCR5*Δ*32 polymorphism in the Turkmen population of Golestan province, northeast of Iran. Blood samples were collected from 400 randomly selected Turkmen populations (199 women and 201 men), and genomic DNA was extracted. Characterization of CCR5*Δ*32 genotypes was performed by PCR using primers flanking the 32-nucleotide deletion in the *CCR5* gene. The amplified DNA fragments were visualized on 2% agarose gel electrophoresis with cybergreen staining under UV light. All individuals were of Turkmen ethnicity and lived in the Golestan province, northeast of Iran. The mean age of all participants was 35.46 years, with a 20-45 year range. All the studied subjects were healthy without any severe conditions such as autoimmune disease and viral infections. All individuals had no history of HIV infection. The PCR product visualization showed that all the samples are at the 330 bp size, which means the CCR5*Δ*32 allele was utterly absent from the study population. The presence of the CCR5*Δ*32 allele among Turkmens may be attributed to the admixture with European descent people. We conclude that the CCR5*Δ*32 polymorphism may be absent in the Iranian Turkmen population, and further studies with a large population are needed.

## 1. Introduction

The chemokine receptor (C-C motif) 5 (CCR5), also known as CD195, is a heptahelical surface protein belonging to the superfamily of GPCRs (G-protein coupled receptors), which cognate chemokine (C-C motif) ligands such as CCL5, CCL3, CCL4, CCL2, and CCL3L1 [1, 2]. CCR5 is expressed in nonhematopoietic peripheral tissues, the central nervous system (CNS), and a vast array of bone marrow-derived cells, including T lymphocytes, monocyte/macrophages, granulocytes, dendritic cells, and natural killer cells [3]. The receptor plays a vital role in the host defense mechanism and inflammation by recruiting immune cells via directing chemotaxis (cell migration) along the chemokine gradient [4, 5]. Moreover, the receptor acts as a learning, plasticity, and memory suppressor [6] and closes the temporal window for memory linking [7].

CCR5*Δ*32 (rs333) is a 32-base pair deletion in the coding region of the *CCR5* gene on the human chromosome 3, which results in a frameshift in the protein sequence leading to the expression of truncated CCR5 and aborting its localization on the cell surface [8, 9]. CCR5*Δ*32 heterozygous (WT/*Δ*32) individuals have shown a decreased expression of functional CCR5 on the cell surface compared to CCR5 wild-type cells. However, people with homozygous CCR5*Δ*32 (*Δ*32/*Δ*32) have no CCR5 on their plasmatic membrane [10–12]. The most abundant CCR5*Δ*32 allele frequency is observed in the Caucasian population (European descent) (~10%), while this allele is nearly absent in Africans, Native Americans, and Asians [13–15]. In the Iranian population, the frequency of CCR5*Δ*32 ranges from 0.0033 to 1.6 due to the diversity of ethnicities in Iran [16, 17].

It is well known that the CCR5*Δ*32 genetic variation could affect some human diseases. The human immunodeficiency virus 1 (HIV-1) needs the CD4 receptor and at least one coreceptor, usually CCR5, for entry or infectivity of cells [18]. As the CCR5*Δ*32 heterozygous genotype (WT/*Δ*32) promotes a decreased expression of functional CCR5 on the cell surface, this genotype has slight protection against HIV infection progression. On the other hand, since there is no CCR5 on the cell membrane of people with homozygous genotype, this polymorphism (*Δ*32/*Δ*32) shows strong protection against HIV-1 infection [19, 20]. CCR5*Δ*32 genetic variation also leads to a protective effect on *Streptococcus pneumoniae* [21], *Staphylococcus aureus* [22], Dengue virus [23], and a severe form of coronavirus disease 2019 (COVID-19) [24]. In contrast, the CCR5*Δ*32 polymorphism is associated with the disease severity of West Nile Virus (WNV) [25], influenza virus [26], tick-borne encephalitis (TBE) [27], and chronic hepatitis B virus (HBV) infection [28]. Additionally, CCR5*Δ*32 is related to impaired brain function, atherosclerosis development [29, 30], and many inflammatory and autoimmune diseases, such as rheumatoid arthritis (RA) [31] and SLE [32]. Here, we aimed to evaluate the prevalence of CCR5*Δ*32 polymorphism in the Turkmen population of Golestan province, northeast of Iran. The population of Golestan province consists of different ethnic groups such as the Fars, Mazni, Azeri, Baloch, Sistani Persians, and Turkmen [33]. About 1.5 million Turkmen live in Iran, and this population mainly lives in the northeast of the country, which is located near the border of Iran-Turkmenistan. Turkmens value their traditions and cultural roots very highly, have preserved them in their families, and have their way of life and customs [34]. Moreover, studying populations using molecular techniques is very important and helpful for their characterization [35, 36]. Conservation of genetic diversity in population requires the proper performance of conservation superiorities and sustainable handling plans based on universal information on population structures, including genetic diversity resources among and between populations [37, 38]. Genetic diversity is essential for genetic improvement, preserving populations, evolution, and adapting to variable environmental situations [39, 40]. On the other hand, determining gene polymorphism is essential in populations [41, 42] to define genotypes and their associations with health and performance [43–45]. Hence, we aimed to evaluate the prevalence of CCR5*Δ*32 polymorphism in the Turkmen population of Golestan province, northeast of Iran.

## 2. Materials and Methods

**Figure 1:**
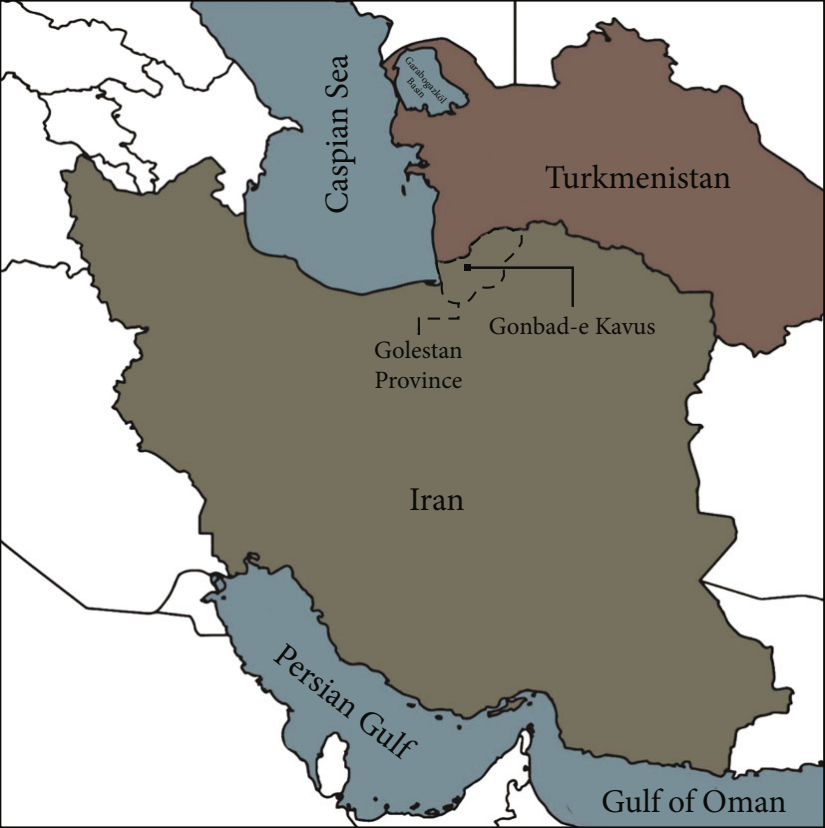
The geographical location of Golestan province, Iran. The majority of the Turkmen population of Iran lives in Golestan province, which are mainly scattered in the cities of Gonbad-e Kavus, Bandar-e Turkman, Gorgan, and Turkmen Sahara.

This cross-sectional study was performed on 400 randomly selected Turkmen populations (199 women and 201 men) from different laboratories in Gonbad-e Kavus, Golestan, Iran. The mean age of all participants was 35.46 years, with a 20-45 year range. The sample size was calculated considering the frequency of this allele in northeastern Iran and the Turkmen population in Iran. The population living in Golestan province consists of many races and ethnicities, such as Fars, Turks, and Turkmen. Unlike the previous study, which was conducted in the same province on different ethnicities [46], the inclusion criteria of this study were the Turkmen ethnicity of the Turkmen parents and grandparents. All individuals were of the exact geographical origin with Turkmen ethnicity, and none were related. The geographical location of the Golestan province is shown in Figure 1. All subjects were informed of the purpose of the study, and informed consent was obtained from all participants. The current study was approved by the Science and Bioethics Committee of Golestan University of Medical Sciences (IR.GOUMS.REC.1400.332).

Blood samples were aseptically collected via venipuncture from each study participant into sterile vacutainer tubes containing ethylenediaminetetraacetic acid (EDTA) anticoagulant. Following collection, the samples were immediately transferred to a refrigerated environment set to a temperature of 4°C until processed for DNA extraction. The genomic DNA was extracted following the manufacturers' instructions (Pioneer, Pishgam, Iran). Spectrophotometric analysis was performed (including optical density (OD) 260/OD 280 and OD 260/OD 230 measurements) to determine the concentration and quality of the extracted DNA (DeNovix Inc., USA). Characterization of CCR5*Δ*32 genotypes was performed by polymerase chain reaction (PCR) using forward primer 5′-TCCTGACAATCGATAGGTACCTGGCT-3′ and reverse primer 5′-GCCTCTTCTTCTCATTTCGACACCGA-3′ flanking the 32-nucleotide deletion in the *CCR5* gene which was designed by Donyavi et al. [47]. The primers were used to amplify the 330 bp segment of the wild-type allele and the 298 bp segment of the mutant allele of CCR5. PCR amplification was carried out in a 20 *μ*l reaction containing 1x Amplicon PCR Master Mix, 250 ng of extracted genomic DNA, and 0.5 *μ*M of each primer. The thermal cycle profiles were as follows: initial denaturation for 6 minutes at 94°C and 35 cycles of 94°C for 35 seconds, 65°C for 40 seconds, and 72°C for 45 seconds, followed by a final extension at 72°C for 5 minutes. Finally, the DNA fragments were visualized on 2% agarose gel electrophoresis with cybergreen staining under UV light.

## 3. Results

A total of 400 people participated in this study, consisting of 201 men (50.2%) and 199 women (49.8%). The mean age of female and male subjects was 34.86 and 36.54 years, respectively. All the studied subjects were healthy, and based on the completed questionnaires, they had no severe conditions such as autoimmune disease and viral infections. All individuals had no history of HIV infection. The details of the demographic data are presented in Table 1.

Our results indicate that the extracted DNA samples had high purity levels. Specifically, all samples yielded values above the acceptable threshold of 1.5 for the ratio absorbance of 280/260, indicating the absence of contaminants such as RNA or other organic substances. We examined the optical absorption ratio of 260/230 to evaluate protein contamination, which also yielded favorable results. Specifically, all samples had values within the acceptable range of 2.0 to 2.2, indicating the absence of protein contaminants. These findings demonstrate the effectiveness of our DNA extraction method in isolating high-quality DNA samples for downstream applications.

**Figure 2:**
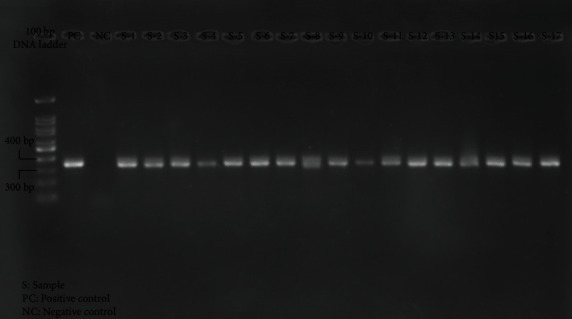
CCR5 genotype determination by agarose gel electrophoresis. Lane 1: 100 bp DNA ladder. Lane 2: positive control (PC). Lane 3: negative control (NC). Other lanes: wild-type genotype (CCR5/CCR5).

The presence of CCR5 genetic variations was investigated in all samples using the PCR method via specific primers. The primers are designed to amplify the 330 bp segment of the wild-type allele and the 298 bp segment of the mutant allele of CCR5. The PCR product visualization on 2% agarose gel electrophoresis with cybergreen staining under UV light showed that all the samples are at the 330 bp size, which means the CCR5*Δ*32 allele was utterly absent from the study population. The PCR results of 17 samples with positive and negative controls are shown in Figure 2.

## 4. Discussion

The frequency of the CCR5*Δ*32 allele in the world is approximately equal to 3% [48]. This allele is mainly present in European countries (about 10%). The highest frequency of this allele has been reported in the surrounding area of the Baltic and White Sea and the central regions of Russia (>15%) [49]. On the other hand, this polymorphism is very rare in North Africa, the Middle East, and Central Asia. Also, CCR5*Δ*32 is absent in East and Southeast Asian populations, natives of America, Oceania, and sub-Saharan Africa [50]. In the Iranian people, the frequency of CCR5*Δ*32 is estimated at 1.6%, which varies in different ethnicities living in this country [16, 17]. By the end of 2021, 38.4 million HIV-infected individuals worldwide have been detected, with the highest frequency observed in South and West Africa (20.6 million infected) and the lowest in the Middle East and North Africa (18,000 infected) [51]. By the end of 2018, 38,996 people were diagnosed with HIV infection in Iran [52].

In the present study, the frequency of the CCR5*Δ*32 allele in healthy Turkmen people was not found. This is consistent with other studies from Iran [16, 17, 46, 53, 54] and other countries [55–62]. In contrast to our research, Trecarichi et al. have shown a significantly higher frequency of *Δ*32 in the healthy control group compared to HIV-positive people. Moreover, Philpott et al. reported a two-fold higher frequency of the *Δ*32 genotype in the healthy control compared with HIV-1-seropositive people. Many factors contribute to this diversity report, such as variations in ethnicity, which may play a significant role in the population. Multiple linear regression analyses by Thomas et al., adjusted for age and race, showed a significant negative association between HIV risk duration and CCR5 expression on monocytes [63]. Another study by Meditz et al. showed that CCR5 expression is reduced in the lymph nodes of HIV-1-infected women compared with men but does not mediate sex-based differences in viral loads [64].

The discussed origin of the CCR5*Δ*32 allele is still controversial. Beyond the proposed theories, Sabeti et al. indicated that this polymorphism became frequent in the European population due to neutral evolution. However, the authors have suggested the possible role of selective pressure in the historical period as an element in CCR5*Δ*32 frequency [65, 66]. The racial distribution of HIV risk raises the possibility that differences in the distribution of the CCR5*Δ*32 allele or other heritable host factors/mutations may influence the rate of transmission or the speed of the epidemic in different racial groups [67]. Genetically, Iranians are considered close to North Indians, Greeks, and specific European populations such as Italians, Germans, and British, which can be related to the following factors: (1) Indo-European same ancestor population (Aryans); (2) significant genetic admixture of Iranians with their neighbors in different eras; and (3) connecting the eastern and western Eurasian populations through the Silk Road network, which led to migrations and genetic admixture along this network [53]. Recent data on allele and haplotype frequencies of human leukocyte antigen (HLA) class II have confirmed the similarity in the genetic ancestry of Iranians, Greeks, and Italians [68, 69]. Historical evidence suggests differences between the population of Iran and Europeans of the same ancestry, known as Indo-Europeans. Around 2000 BC, an Indo-European tribe named Aryans invaded central Asia and occupied Iran, Iraq, the north of India, and Afghanistan. The significant difference between the western and eastern migrations of Indo-Europeans is that they mixed genetically with similar populations in the west. In contrast, in the east, they mixed with others. Genetically, together have produced a primarily mixed population, and the Arabs gradually diluted the primary Indo-Aryan traces over the centuries [69]. The rate of progression to AIDS varies among individuals infected with HIV-1, and it has been shown that CCR5Δ32 confers almost complete resistance to HIV-1 infection in homozygotes and partial protection against HIV disease progression in heterozygous adults.

CCR5 (formerly CKR5) plays a crucial role in the chemotaxis of immune cells to inflammation sites and in mediating inflammatory responses. Beyond cell migration, CCR5 is involved in the surveillance of the immune cells, inflammation, and the pathogenesis of inflammatory diseases and cancers [3]. CCR5*Δ*32 is a 32 bp deletion in the *CCR5* gene on the second extracellular loop encoding region. This variation results in a frameshift in the CCR5 protein sequences, leading to a prematurely truncated protein lacking three transmembrane domains. CCR5*Δ*32 mutant protein has no function and does not localize on the cell surface [13, 70]. The CCR5*Δ*32 has received much attention due to its protective role against HIV infection and disease progression [20]. Beyond HIV infection, data has shown that this polymorphism is correlated to other conditions such as autoimmune diseases and pathogenic infections [3, 71].

A protective effect of CCR5*Δ*32 allele variation on some conditions such as childhood asthma [72], type 2 diabetes (noninsulin-dependent diabetes mellitus) [73], hepatitis B virus (HBV) [74], liver inflammation in HCV infection [75, 76], osteomyelitis of *Staphylococcus aureus* infection [77], and toxoplasmosis [78, 79] has been reported. On the other hand, this polymorphism has been considered a risk factor for multiple sclerosis (MS) [80], symptomatic West Nile virus (WNV) infection [81], the severity of influenza virus [26], severe form of coronavirus disease-2019 (COVID-19) [24], *Streptococcus pneumoniae* infection [21], and tuberculosis [82].

The gradient distribution of the CCR5*Δ*32 polymorphism from north to south with the highest frequency in the Nordic population suggests a Scandinavian origin of this polymorphism. Subsequently, this variant spread to the European population through raids of the Vikings in the 8th-10th centuries [66, 83]. The CCR5*Δ*32 allele frequency around the world is estimated at approximately 3% based on the information obtained from various studies on more than 5000 samples [84, 85]. This genetic variation is primarily observed in Caucasian populations (of European ethnicity), where the average mutant allele frequency is about 10% of this population [13]. In contrast to the European population, CCR5*Δ*32 polymorphism is virtually absent in Asians, Sub-Saharan Africans, and Native American ethnicities [15]. The CCR5*Δ*32 allele frequency in Asian countries and Iran is about 2.06% of the population [17].  Table 2 displays the outcomes of prior investigations conducted on various populations and ethnicities residing in Iran, as well as the findings from our current study. This study is aimed at determining the distribution of the CCR5*Δ*32 polymorphism in the Turkmen population in the north of Iran. The CCR5*Δ*32 allele was utterly absent from our study population. In this study, we had a limitation on sample size and no access to HIV-infected patients in the Turkmen population. Also, due to the absence of mutant alleles (homozygous or heterozygous) among this study's samples, it was impossible to calculate the Hardy-Weinberg equilibrium. Further studies with large sample size are needed to investigate the frequency of CCR5 mutations and its association with diseases.

## 5. Conclusion

In conclusion, the recent study confirms that the CCR5*Δ*32 polymorphism is absent in the Turkmen population residing in northern Iran. The absence of this polymorphism is expected to persist in this community due to their cultural practices, regardless of whether its evolution was driven by environmental pressure or neutral evolution across various populations over decades.

## Figures and Tables

**Table 1:** The demographic and clinical characteristics of participants.

	Male	Female	Total
Individuals	201	199	400
Mean of age (years)	36.54	34.86	35.46
Median of age (years)	38	35	37
Ethnicity	Turkmen	Turkmen	Turkmen
HIV/AIDS	Negative	Negative	Negative
Other viral infections^∗^	Negative	Negative	Negative
Autoimmune disease	No background	No background	No background
History of HIV infection	No background	No background	No background
CCR5 genotype	WT-WT (100%)	WT-WT (100%)	WT-WT (100%)

^∗^HBV, HCV, SARS-CoV-2, influenza A, and B.

**Table 2:** CCR5*Δ*32 allele frequency (%) distribution in the Iranian population living in different geographical locations.

Population	Sample size	Genotypes (%)			*Δ*32 allele frequency	Reference
		WT/WT	WT/*Δ*32	*Δ*32/*Δ*32		
Turkmen (north of Iran)	400	400 (100)	0 (0)	0 (0)	0%	Current study
Fars province (south of Iran)	395	384 (97.2)	11 (2.8)	0 (0)	1.4%	[53]
Urmia city (northwest of Iran)	200	186 (97.89)	4 (2.105)	0 (0)	1.05%	[54]
Mashhad city (northeast of Iran)	400	388 (97)	11 (2.75)	1 (0.25)	1.6%	[16]
Golestan province (southeast of the Caspian Sea)	300	291 (97)	9 (3)	0 (0)	1.5%	[46]
Tehran province (capital of Iran)	371	357 (96.2)	14 (3.8)	0 (0)	3.8%	[47]
Iran	530	523 (98.5)	6 (1.1)	1 (0.19)	1.1%	[17]

## Data Availability

The datasets during and/or analyzed during the current study are available from the corresponding author on reasonable request.

## Abbreviations

GPCR: G-protein coupled receptors
CCR5: The chemokine receptor (C–C motif) 5
RANTES: Regulated on activation, normal T cell expressed and secreted
MIP-1*α*/*β*: Macrophage inflammatory protein-1 alpha/beta
MCP-1: Monocyte chemoattractant protein-1
CCL3L1: Chemokine (C-C motif) ligand 3-like 1
DCs: Dendritic cells
NK cells: Natural killer cells
HIV-1: Human immunodeficiency virus 1
WNV: West Nile virus
HBV: Hepatitis B virus
RA: Rheumatoid arthritis
SLE: Systemic lupus erythematosus
NIDDM: Noninsulin-dependent diabetes mellitus.

## References

[1] Scholten D. J., Canals M., Maussang D. (2012). Pharmacological modulation of chemokine receptor function. *British Journal of Pharmacology*.

[2] Miyakawa T., Obaru K., Maeda K., Harada S., Mitsuya H. (2002). Identification of amino acid residues critical for LD78*β*, a variant of human macrophage inflammatory protein-1*α*, binding to CCR5 and inhibition of R5 human immunodeficiency virus type 1 replication. *Journal of Biological Chemistry*.

[3] Jasinska A. J., Pandrea I., Apetrei C. (2022). CCR5 as a coreceptor for human immunodeficiency virus and simian immunodeficiency viruses: a prototypic love-hate affair. *Frontiers in Immunology*.

[4] Moreira A. P., Cavassani K. A., Tristão F. S. M. (2008). CCR5-dependent regulatory T cell migration mediates fungal survival and severe Immunosuppression. *Journal of Immunology*.

[5] Luangsay S., Kasper L. H., Rachinel N. (2003). CCR5 mediates specific migration of *Toxoplasma gondii*--primed CD8^+^ lymphocytes to inflammatory intestinal epithelial cells. *Gastroenterology*.

[6] Zhou M., Greenhill S., Huang S. (2016). CCR5 is a suppressor for cortical plasticity and hippocampal learning and memory. *Elife*.

[7] Shen Y., Zhou M., Cai D. (2022). CCR5 closes the temporal window for memory linking. *Nature*.

[8] Liu X., Kim C. N., Yang J., Jemmerson R., Wang X. (1996). Induction of apoptotic program in cell-free extracts: requirement for dATP and cytochrome c. *Cell*.

[9] Dean M., Carrington M., Winkler C. (1996). Genetic restriction of HIV-1 infection and progression to AIDS by a deletion allele of the CKR5 structural gene. *Science*.

[10] Dragic T., Litwin V., Allaway G. P. (1996). HIV-1 entry into CD4^+^ cells is mediated by the chemokine receptor CC-CKR-5. *Nature*.

[11] Venkatesan S., Petrovic A., Van Ryk D. I., Locati M., Weissman D., Murphy P. M. (2002). Reduced cell surface expression of CCR5 in CCR5*Δ*32 heterozygotes is mediated by gene dosage, rather than by receptor sequestration. *Journal of Biological Chemistry*.

[12] Picton A. C. P., Shalekoff S., Paximadis M., Tiemessen C. T. (2012). Marked differences in CCR5 expression and activation levels in two south African populations. *Immunology*.

[13] Samson M., Libert F., Doranz B. J. (1996). Resistance to HIV-1 infection in Caucasian individuals bearing mutant alleles of the CCR-5 chemokine receptor gene. *Nature*.

[14] Dean M., Carrington M., Winkler C. (1996). Genetic restriction of HIV-1 infection and progression to AIDS by a deletion allele of the CKR5 structural gene. Hemophilia Growth and Development Study, Multicenter AIDS Cohort Study, Multicenter Hemophilia Cohort Study, San Francisco City Cohort, ALIVE Study. *Science*.

[15] Stephens J. C., Reich D. E., Goldstein D. B. (1998). Dating the origin of the *CCR5-Δ32* AIDS-resistance allele by the coalescence of haplotypes. *American Journal of Human Genetics*.

[16] Tajbakhsh A., Fazeli M., Rezaee M. (2019). Prevalence of *CCR5delta32* in northeastern Iran. *BMC Medical Genetics*.

[17] Rahimi H., Farajollahi M. M., Hosseini A. (2014). Distribution of the mutated delta 32 allele of CCR5 co-receptor gene in Iranian population. *Medical Journal of the Islamic Republic of Iran*.

[18] Cicala C., Arthos J., Fauci A. S. (2011). HIV-1 envelope, integrins and co-receptor use in mucosal transmission of HIV. *Journal of Translational Medicine*.

[19] Proudfoot A. E. I. (2002). Chemokine receptors: multifaceted therapeutic targets. *Nature Reviews Immunology*.

[20] Ellwanger J. H., Kulmann-Leal B., Kaminski V. . L., Rodrigues A. G., Bragatte M. A. . S., Chies J. A. B. (2020). Beyond HIV infection: neglected and varied impacts of CCR5 and CCR5*Δ*32 on viral diseases. *Virus Research*.

[21] Salnikova L. E., Smelaya T. V., Moroz V. V., Golubev A. M., Rubanovich A. V. (2013). Host genetic risk factors for community-acquired pneumonia. *Gene*.

[22] Alonzo F., Kozhaya L., Rawlings S. A. (2013). CCR5 is a receptor for *Staphylococcus aureus* leukotoxin ED. *Nature*.

[23] Marques R. E., Guabiraba R., Del Sarto J. L. (2015). Dengue virus requires the CC-chemokine receptor CCR5 for replication and infection development. *Immunology*.

[24] Starcevic Cizmarevic N., Kapovic M., Roncevic D., Ristic S. (2021). Could the CCR5-*Δ*32 mutation be protective in SARS-CoV-2 infection?. *Physiological Research*.

[25] Glass W. G., Lim J. K., Cholera R., Pletnev A. G., Gao J. L., Murphy P. M. (2005). Chemokine receptor CCR5 promotes leukocyte trafficking to the brain and survival in West Nile virus infection. *The Journal of Experimental Medicine*.

[26] Falcon A., Cuevas M. T., Rodriguez-Frandsen A. (2015). CCR5 deficiency predisposes to fatal outcome in influenza virus infection. *The Journal of General Virology*.

[27] Ellwanger J. H., Chies J. A. B. (2019). Host immunogenetics in tick-borne encephalitis virus infection--The CCR5 crossroad. *Ticks and Tick-borne Diseases*.

[28] Suneetha P. V., Sarin S. K., Goyal A., Kumar G. T., Shukla D. K., Hissar S. (2006). Association between vitamin D receptor, CCR5, TNF-*α* and TNF-*β* gene polymorphisms and HBV infection and severity of liver disease. *Journal of Hepatology*.

[29] Ellwanger J. H., de L Kaminski V., Chies J. A. B. (2020). What we say and what we mean when we say redundancy and robustness of the chemokine system – how CCR5 challenges these concepts. *Immunology & Cell Biology*.

[30] Ellwanger J. H., Kaminski V. . L., Chies J. A. B. (2019). *CCR5* gene editing - Revisiting pros and cons of CCR5 absence. *Infection, Genetics and Evolution*.

[31] Cooke S. P., Forrest G., Venables P. J., Hajeer A. (1998). The *Δ*32 deletion of CCR5 receptor in rheumatoid arthritis. *Arthritis and Rheumatism*.

[32] Carvalho C., Calvisi S. L., Leal B. (2014). CCR5-Delta32: implications in SLE development. *International Journal of Immunogenetics*.

[33] Ameryoun A., Meskarpour-Amiri M., Dezfuli-Nejad M. L., Khoddami-Vishteh H., Tofighi S. (2011). The assessment of inequality on geographical distribution of non-cardiac intensive care beds in Iran. *Iranian Journal of Public Health*.

[34] Irons W. (1969). The Turkmen of Iran: a brief research report. *Iranian Studies*.

[35] Mohammadi A., Nassiry M., Mosafer J., Mohammadabadi M., Sulimova G. (2009). Distribution of *BoLA-DRB3* allelic frequencies and identification of a new allele in the Iranian cattle breed Sistani (*Bos indicus*). *Russian Journal of Genetics*.

[36] Mohammadabadi M. (2021). Tissue-specific mRNA expression profile of ESR2 gene in goat. *Agricultural Biotechnology Journal*.

[37] Javanmard A., Mohammadabadi M., Zarrigabayi G. (2008). Polymorphism within the intron region of the bovine leptin gene in Iranian Sarabi cattle (Iranian Bos taurus). *Russian Journal of Genetics*.

[38] Roudbar M. A., Abdollahi-Arpanahi R., Mehrgardi A. A., Mohammadabadi M., Yeganeh A. T., Rosa G. (2018). Estimation of the variance due to parent-of-origin effects for productive and reproductive traits in Lori-Bakhtiari sheep. *Small Ruminant Research*.

[39] Mousavizadeh A., Mohammad Abadi M., Torabi A., Nassiry M. R., Ghiasi H., AliEsmailizadeh Koshkoieh A. (2009). Genetic polymorphism at the growth hormone locus in Iranian Talli goats by polymerase chain reaction-single strand conformation polymorphism (PCR-SSCP). *Iranian Journal of Biotechnology*.

[40] Masoudzadeh S., Mohammadabadi M., Khezri A. (2020). Dlk1 gene expression in different tissues of lamb. *Iranian Journal of Applied Animal Science*.

[41] Mohammadabadi M., Soflaei M., Mostafavi H., Honarmand M. (2011). Using PCR for early diagnosis of bovine leukemia virus infection in some native cattle. *Genetics and Molecular Research*.

[42] Ahsani M., Mohammadabadi M., Shamsaddini M. (2010). Clostridium perfringens isolate typing by multiplex PCR. *Journal of Venomous Animals and Toxins Including Tropical Diseases*.

[43] Nassiry M., Eftekhar Shahroodi F., Mosafer J. (2005). Analysis and frequency of bovine lymphocyte antigen (BoLA-DRB3) alleles in Iranian Holstein cattle. *Russian Journal of Genetics*.

[44] Norouzy A., Nassiry M. R., Eftekhari Shahrody F., Javadmanesh A., Mohammad Abadi M. R., Sulimova G. E. (2005). Identification of bovine leucocyte adhesion deficiency (BLAD) carriers in Holstein and Brown Swiss AI bulls in Iran. *Russian Journal of Genetics*.

[45] Sulimova G., Azari M. A., Rostamzadeh J., Mohammad Abadi M. R., Lazebny O. E. (2007). *κ*-casein gene (*CSN3*) allelic polymorphism in Russian cattle breeds and its information value as a genetic marker. *Russian Journal of Genetics*.

[46] Heydarifard Z., Tabarraei A., Moradi A. (2017). Polymorphisms in CCR5*Δ*32 and risk of HIV-1 infection in the southeast of Caspian Sea, Iran. *Disease Markers*.

[47] Donyavi T., Bokharaei-Salim F., Nahand J. S. (2020). Evaluation of CCR5-*Δ*32 mutation among individuals with high risk behaviors, neonates born to HIV-1 infected mothers, HIV-1 infected individuals, and healthy people in an Iranian population. *Journal of Medical Virology*.

[48] E release 1000 Genomes Project Phase 3 allele frequencies rs333. https://www.ensembl.org/Homo_sapiens/Variation/Population?db=core;r=3:46372953-46373987;v=rs333;vdb=variation;vf=90066634.

[49] Balanovsky O., Pocheshkhova E., Pshenichnov A. (2005). Is spatial distribution of the HIV-1-resistant CCR5*Δ*32 allele formed by ecological factors?. *Journal of Physiological Anthropology and Applied Human Science*.

[50] Faure E., Royer-Carenzi M. (2008). Is the European spatial distribution of the HIV-1-resistant CCR5-*Δ*32 allele formed by a breakdown of the pathocenosis due to the historical Roman expansion?. *Infection, Genetics and Evolution*.

[51] (2022). *Global HIV & AIDS Statistics — Fact Sheet*.

[52] SeyedAlinaghi S., Taj L., Mazaheri-Tehrani E. (2021). HIV in Iran: onset, responses, and future directions. *AIDS*.

[53] Gharagozloo M., Doroudchi M., Farjadian S., Pezeshki A. M., Ghaderi A. (2005). The frequency of CCR5*Δ*32 and CCR2-64I in southern Iranian normal population. *Immunology Letters*.

[54] Omrani D. (2009). Frequency of CCR5? 32 variant in north-west of Iran. *Journal of Sciences, Islamic Republic of Iran*.

[55] Gomulska M., Rusin G., Gwiazdak P. (2014). Prevalence of CCR5-delta32 mutation in asthmatic and non-asthmatic subjects from department of medicine, JUCM, Cracow. *Folia Medica Cracoviensia*.

[56] Ferreira-Fernandes H., Santos A. C. C., Motta F. J. N. (2015). Prevalence of *CCR5*-*Δ*32 and *CCR2*-V64I polymorphisms in a mixed population from northeastern Brazil. *Genetics and Molecular Research*.

[57] Köksal M. O., Akgül B., Beka H. (2021). Frequency of CCR5-*Δ*32, CCR2-64I and SDF1-3′A alleles in HIV-infected and uninfected patients in Istanbul, Turkey. *The Journal of Infection in Developing Countries*.

[58] Bharti D., Kumar A., Mahla R. S. (2015). Low prevalence of CCR5-*Δ*32, CCR2-64I and SDF1-3′A alleles in the Baiga and Gond tribes of Central India. *Springerplus*.

[59] Hütter G., Blüthgen C., Elvers-Hornung S., Klüter H., Bugert P. (2015). Distribution of the CCR5-delta32 deletion in Southwest Germany. *Anthropologischer Anzeiger*.

[60] Zheng B., Wiklund F., Gharizadeh B. (2006). Genetic polymorphism of chemokine receptors CCR2 and CCR5 in Swedish cervical cancer patients. *Anticancer Research*.

[61] Novembre J., Galvani A. P., Slatkin M. (2005). The geographic spread of the CCR5 *Δ*32 HIV-resistance Allele. *PLoS Biology*.

[62] Melum E., Karlsen T. H., Broome U. (2006). The 32-base pair deletion of the chemokine receptor 5 gene (*CCR5-Δ32*) is not associated with primary sclerosing cholangitis in 363 Scandinavian patients. *Tissue Antigens*.

[63] Thomas S. M., Tse D. B., Ketner D. S. (2006). CCR5 expression and duration of high risk sexual activity among HIV-seronegative men who have sex with men. *AIDS*.

[64] Meditz A. L., Folkvord J. M., Lyle N. H. (2014). CCR5 expression is reduced in lymph nodes of HIV type 1-infected women, compared with men, but does not mediate sex-based differences in viral Loads. *The Journal of Infectious Diseases*.

[65] Ellwanger J. H., Kaminski V. L., Rodrigues A. G., Kulmann-Leal B., Chies J. A. B. (2020). CCR5 and CCR5*Δ*32 in bacterial and parasitic infections: thinking chemokine receptors outside the HIV box. *International Journal of Immunogenetics*.

[66] Sabeti P. C., Walsh E., Schaffner S. F. (2005). The case for selection at *CCR5-Δ32*. *PLoS Biology*.

[67] Salem A. H., Batzer M. A. (2007). Distribution of the HIV resistance CCR5-*Δ*32 allele among Egyptians and Syrians. *Mutation Research*.

[68] Amirzargar A., Mytilineos J., Farjadian S. (2001). Human leukocyte antigen class II allele frequencies and haplotype association in Iranian normal population. *Human Immunology*.

[69] Nei M., Roychoudhury A. K. (1993). Evolutionary relationships of human populations on a global scale. *Molecular Biology and Evolution*.

[70] Guerini F. R., Delbue S., Zanzottera M. (2008). Analysis of CCR5, CCR2, SDF1 and RANTES gene polymorphisms in subjects with HIV-related PML and not determined leukoencephalopathy. *Biomedicine & Pharmacotherapy*.

[71] Ghorban K., Dadmanesh M., Hassanshahi G. (2013). Is the CCR5 *Δ* 32 mutation associated with immune system-related diseases?. *Inflammation*.

[72] Srivastava P., Helms P., Stewart D., Main M., Russell G. (2003). Association of CCR5*Δ*32 with reduced risk of childhood but not adult asthma. *Thorax*.

[73] Muntinghe F. L., Gross S., Bakker S. J. L. (2009). CCR5*Δ*32 genotype is associated with outcome in type 2 diabetes mellitus. *Diabetes Research and Clinical Practice*.

[74] Thio C. L., Astemborski J., Bashirova A. (2007). Genetic protection against hepatitis B virus conferred by *CCR5Δ32*: evidence that CCR5 contributes to viral persistence. *Journal of Virology*.

[75] Goulding C., McManus R., Murphy A. (2005). The CCR5-*Δ*32 mutation: impact on disease outcome in individuals with hepatitis C infection from a single source. *Gut*.

[76] Wald O., Pappo O., Ari Z. B. (2004). The CCR5*Δ*32 allele is associated with reduced liver inflammation in hepatitis C virus infection. *European Journal of Immunogenetics*.

[77] de Souza C. A., de Souza C. A., Cunha L. M. P., de Souza A. Q. A., de Morais M. S., Rabenhorst S. H. B. (2015). A new look at osteomyelitis development - Focus on *CCR5*delta32. Study in patients from Northeast Brazil. *Infection, Genetics and Evolution*.

[78] de Faria Junior G. M., Ayo C. M., de Oliveira A. P. (2018). *CCR5 chemokine receptor* gene polymorphisms in ocular toxoplasmosis. *Acta Tropica*.

[79] Ashton L., Stewart G., Biti R., Law M., Cooper D., Kaldor J. (2002). Heterozygosity for *CCR5-DΔ*32 but not *CCR2b-64I* protects against certain intracellular pathogens. *HIV Medicine*.

[80] Pulkkinen K., Luomala M., Kuusisto H. (2004). Increase in CCR5 *Δ*32/*Δ*32 genotype in multiple sclerosis. *Acta Neurologica Scandinavica*.

[81] Bigham A. W., Buckingham K. J., Husain S. (2011). Host genetic risk factors for West Nile virus infection and disease progression. *PLoS One*.

[82] Mamtani M., Mummidi S., Ramsuran V. (2011). Influence of variations in *CCL3L1* and CCR5 on tuberculosis in a northwestern Colombian population. *Journal of Infectious Diseases*.

[83] Libert F., Cochaux P., Beckman G. (1998). The *Δccr5* mutation conferring protection against HIV-1 in Caucasian populations has a single and recent origin in northeastern Europe. *Human Molecular Genetics*.

[84] Cunningham F., Allen J. E., Allen J. (2022). Ensembl 2022. *Nucleic Acids Research*.

[85] rs333 genetic distribution. https://asia.ensembl.org/Homo_sapiens/Variation/Explore?db=core;r=3:46372953-46373987;v=rs333;vdb=variation;vf=90066634.

